# Transcriptome Analysis Reveals a Potential Role of Benzoxazinoid in Regulating Stem Elongation in the Wheat Mutant *qd*

**DOI:** 10.3389/fgene.2021.623861

**Published:** 2021-02-09

**Authors:** Daxing Xu, Yongdun Xie, Huijun Guo, Weiwei Zeng, Hongchun Xiong, Linshu Zhao, Jiayu Gu, Shirong Zhao, Yuping Ding, Luxiang Liu

**Affiliations:** National Engineering Laboratory for Crop Molecular Breeding, Institute of Crop Sciences, Chinese Academy of Agricultural Sciences, National Center of Space Mutagenesis for Crop Improvement, Beijing, China

**Keywords:** transcriptome, benzoxazinoids, stem elongation, mutant, wheat

## Abstract

The stems of cereal crops provide both mechanical support for lodging resistance and a nutrient supply for reproductive organs. Elongation, which is considered a critical phase for yield determination in winter wheat (*Triticum aestivum* L.), begins from the first node detectable to anthesis. Previously, we characterized a heavy ion beam triggered wheat mutant *qd*, which exhibited an altered stem elongation pattern without affecting mature plant height. In this study, we further analyzed mutant stem developmental characteristics by using transcriptome data. More than 40.87 Mb of clean reads including at least 36.61 Mb of unique mapped reads were obtained for each biological sample in this project. We utilized our transcriptome data to identify 124,971 genes. Among these genes, 4,340 differentially expressed genes (DEG) were identified between the *qd* and wild-type (WT) plants. Compared to their WT counterparts, *qd* plants expressed 2,462 DEGs with downregulated expression levels and 1878 DEGs with upregulated expression levels. Using DEXSeq, we identified 2,391 counting bins corresponding to 1,148 genes, and 289 of them were also found in the DEG analysis, demonstrating differences between *qd* and WT. The 5,199 differentially expressed genes between *qd* and WT were employed for GO and KEGG analyses. Biological processes, including protein-DNA complex subunit organization, protein-DNA complex assembly, nucleosome organization, nucleosome assembly, and chromatin assembly, were significantly enriched by GO analysis. However, only benzoxazinoid biosynthesis pathway-associated genes were enriched by KEGG analysis. Genes encoding the benzoxazinoid biosynthesis enzymes Bx1, Bx3, Bx4, Bx5, and Bx8_9 were confirmed to be differentially expressed between *qd* and WT. Our results suggest that benzoxazinoids could play critical roles in regulating the stem elongation phenotype of *qd*.

## Introduction

Sessile plants produce a wide variety of chemical compounds to regulate their growth, stress resistance, and environmental response. Among all metabolic chemicals, benzoxazinoids represent a predominant family produced by numerous *Poaceae* family species including maize, rye, and wheat (Frey et al., 2009; Sue et al., 2011), and those compounds can account for 1% or more of the dry weight of crop seedlings (Zhou et al., 2018). Since benzoxazinoids are important compounds in crops, efforts to elucidate their biosynthetic pathway have been undertaken for more than 50 years (Makowska et al., 2015). Benzoxazinoid biosynthesis involves the chloroplast, endoplasmic reticulum, cytoplasm, and vacuole (Dutartre et al., 2012). It was also suggested that benzoxazinoids can be transported among different tissues (Ahmad et al., 2011). The biochemical process of benzoxazinoid synthesis in plants is catalyzed by enzymes encoded by the *Bx* genes *Bx1*-*Bx9*, while all the identified genes in this process have been mapped in maize, rye, and wheat (Makowska et al., 2015). Among the *Bx* genes, *Bx1* is considered to be responsible for the first committed step of benzoxazinoid biosynthesis in chloroplasts (Tzin et al., 2017). Although the BX1 enzymes in maize and wheat exhibit little similarity in their amino acid sequences, they have comparable catalytic properties (Schullehner et al., 2008). The expression of the *Bx1-Bx5* genes was considered to be co-regulated in hexaploid wheat (Nomura et al., 2005).

Benzoxazinoids in plants are traditionally characterized as crucial signaling molecules involved in the defense against herbivorous insects and pathogens (Niemeyer, 2009). Pathogen infection of plants can activate the benzoxazinoid biosynthesis pathway (Huffaker et al., 2011). In addition to pathogenic stress, the biosynthesis of benzoxazinoids can be regulated by a variety of abiotic factors, such as temperature, light conditions, photoperiod, drought, nitrogen status, plant hormones, and cultivation methods (Oikawa et al., 2002; Villagrasa et al., 2006; Niemeyer, 2009; Bakera et al., 2020). Benzoxazinoids are also constitutively produced in maize plants, even in the absence of a pathogen threat (Zhou et al., 2018), and they are observed to accumulate around the emerging roots of maize, possibly reducing pathogen infections (Park et al., 2004). Since there are multiple factors that could affect the biosynthesis of benzoxazinoids, these compounds may also be involved in the regulation of other plant developmental processes.

Wheat (*Triticum aestivum* L.) serves as an important food crop that provides nearly 55% of the carbohydrates and 20% of the calories consumed globally (Pfeifer et al., 2014). Increasing wheat yield potential remains one of the major breeding objectives for ensuring world food security (Foulkes et al., 2011). The stem elongation phase (SEP) is critical for final yield formation and has been proposed as an important site for improving yield potential in wheat (González et al., 2003; Borras-Gelonch et al., 2012). Based on floral development and the abortion process, the SEP can be dissected into seven stages, known as the terminal spikelet stage, white anther stage, green anther stage, yellow anther stage, tipping stage, heading stage, and anthesis stage (Guo et al., 2018a). During the SEP, the plant height increases quickly, and wheat floret primordia undergo a complicated developmental process. During these seven stages, 75 plant growth-associated traits were quantified (Guo et al., 2018b). The elongation of stems is regulated by various chemical components including plant hormones, such as GA and BR (Oliva et al., 2013; Claeys et al., 2014). Therefore, it was suggested that there may be multiple ways to regulate plant growth during the SEP to increase wheat yield potential (Guo et al., 2018b).

Along with the rapid development of sequencing technologies and the rapid decrease in unit price, transcriptomic analysis has become a powerful tool for elucidating the molecular mechanisms responsible for critical plant growth processes (Yu et al., 2016; Curci et al., 2017). Zhang et al. (2014) demonstrated distinct gene activation in wheat in response to pathogen infection. Using global transcriptome analysis, a gene co-expression regulation network and key genes involved in wheat grain development and heterosis were successfully characterized (Liu et al., 2018; Chi et al., 2019).

The wheat quick development mutant was characterized by a quicker stem elongation rate than that of wild-type plants (Zhang et al., 2016). In this study, through transcriptome analysis methods, we demonstrated that benzoxazinoids may play roles in regulating the stem elongation progress. Our results provide a new perspective for manipulating wheat stem growth to achieve better performance.

## Materials and Methods

### Plant Materials and Sample Preparation

The quick development mutant (*qd*) and its wild type Lunxuan987 were the same as previously described (Zhang et al., 2016). Briefly, we irradiated dry seeds of Lunxuan987 with 40 Gy of ^7^Li ion-beam, and screened the mutant exhibiting an altered stem elongation pattern from the M_2_ generation. Every generation of both wild-type and *qd* mutant plants were covered by paper bags during the anthesis period to ensure their self-pollination. The WT and *qd* seeds were sown in the experimental field of the Institute of Crop Sciences of Chinese Academy of Agricultural Sciences with normal cultivation management. Both *qd* and wild-type plants were planted in 20 rows 1.5 m in length. Thirty plants were uniformly planted in each row.

When the plant height difference between WT and *qd* reached its maximum value at the booting stage, the shoot tip meristem parts were collected as samples. We prepared three biological replicates for both wild-type plants (named WT-1, WT-2, and WT-3) and *qd* plants (named qd-1, qd-2, and qd-3) for RNA isolation, and each replicate contained 15 individual stems from five plants with uniform phenotypes.

### RNA Isolation and Library Construction

Total RNA was extracted using the RNeasy® plant Mini Kit (QIAGEN, Germany) according to the manufacturer’s instructions. After the total RNA was extracted, the residual DNA was digested with DNase I (TaKaRa, Japan). Next, the total RNA was purified by an RNA cleanup kit (TIANGEN, China). The purity, concentration, and integrity of the RNA samples were analyzed by the Nanodrop, Qubit 2, and Agilent 2,100 methods, respectively, as described previously (Xiong et al., 2020).

To construct libraries, we first used oligo(dT) magnetic beads to enrich the mRNA, and then the mRNA fragments were processed using a fragmentation buffer. Then, six-mer random primers were used to synthesize first-strand cDNA. Buffer, dNTPs, RNaseH, and DNA polymerase I were used to synthesize second-strand cDNA. AMPure XP breads were employed to purify cDNA. Next, the purified double-stranded cDNA was subjected to end repair, tailing, and ligation to a sequencing linker followed by the use of AMPure XP beads for fragment size selection and PCR enrichment to obtain a cDNA library. The purified double-stranded cDNA was subsequently subjected to terminal repair, dA tailing, and adaptor ligation. A total of six cDNA libraries were constructed.

### Transcriptome Sequencing and Assembly

The libraries were sequenced as 150-bp paired-end reads using the Illumina high-throughput sequencing platform. After the sequencing linker was removed, the primer sequence was removed, and the low-quality reads were filtered out, a total of 53.73 Gb of clean data were obtained. The clean data of each sample were more than 7.49 Gb, and the percentage of Q30 bases was not less than 94.90%. The datasets presented in this study can be found in online repositories. The names of the repositories and accession number can be found at: https://www.ncbi.nlm.nih.gov/sra/; PRJNA682455.

Using the TopHat2 software, clean reads were compared to the wheat reference genome (IWGSC RefSeq v1.0, http://www.wheatgenome.org/). The results from TopHat 2 were spliced using the Cufflinks software, and the stitching results were compared with the original genome annotation information.

### Identification and Annotation of Differentially Expressed Genes and Differential Exon Usage

FPKM (fragments per kilobase of transcript per million fragments mapped) was used as an indicator of transcript or gene expression levels. DEGs were identified using the DESeq2 package. The screening criteria that we used were as follows: the ratio of fold change (FC) was equal to or greater than 2 in the two groups (wild-type vs. *qd*), and the FDR was less than 0.01. To identify differential exon usage (DEUs), the DEXSeq package in R was used for analysis. We used FDR < 0.01 as a screening standard for counting bins to control false positives. After combining the genes from DEGs and DEUs, and setting FDR to less than 0.05, we used OmicShare tools^1^ to detect GO terms and KEGG pathways that were significantly enriched in the genome-wide context. A hypergeometric test was employed to define the significance of enrichment analysis for GO categories.

### Verification of Gene Expression

To confirm the RNA-Seq results, we selected several upregulated and downregulated genes for qRT-PCR validation. After RNA extraction, genomic DNA was removed using DNase I (TaKaRa, Japan) and purified with an RNA clean Kit (TIANGEN, China). Reverse transcription was performed using an iScriptTM cDNA Synthesis Kit (Bio-Rad, America), and SsoFast™ EvaGreen® Supermix reagents (Bio-Rad, America) were used in a Bio-Rad CFX96 real-time fluorescence quantitative PCR instrument. qRT-PCR was performed with the following protocol: 95°C for 3 min, followed by 40 cycles of 95°C for 30 s, 59°C for 30 s, and 72°C for 10 s. Actin was used as a reference gene, and relative quantification was performed using the 2^−ΔΔCt^ method. The primers for actin were as follows: forward, 5'-GTAGGAAATGGCTGACGGTG-3' and reverse, 5'-ATGCTAGGGAAAACAGCCCT-3'. The primers used to verify benzoxazinoid synthesis gene expression are listed in Supplementary Table 1.

## Results

### Clean Reads Data From Wild-Type and *qd* Plants

From the transcriptome sequencing data, we obtained at least 40.87 M of clean reads for each wild-type and *qd* sample. At least 36.61 M of unique mapped reads were detected in our samples, and the unique ratio ranged from 79.47 to 80.95, as listed in Table 1.

**Table 1:** Comparison of clean reads and the reference genome.

Samples	Clean reads	Mapped reads	Unique mapped	Mapped ratio (%)	Unique ratio (%)
WT-1	60,998,726	49,289,966	44,444,399	80.80	72.86
WT-2	82,280,448	65,391,443	59,308,144	79.47	72.08
WT-3	64,259,688	52,015,263	46,484,680	80.95	72.34
qd-1	52,274,004	41,752,122	37,829,434	79.87	72.37
qd-2	53,997,780	43,257,391	38,847,072	80.11	71.94
qd-3	50,797,950	40,867,403	36,613,635	80.45	72.08

Clean reads: the number of reads obtained after quality control. Mapped reads: the number of clean reads aligned to the reference genome. Unique mapped: the number of clean reads aligned to a unique position in the reference genome. Mapped ratio: the number of mapped reads as a percentage of the number of clean reads. Unique ratio: the number of unique mapped reads as a percentage of the number of clean reads.

### Identification of DEGs and DEUs

**Figure 1:**
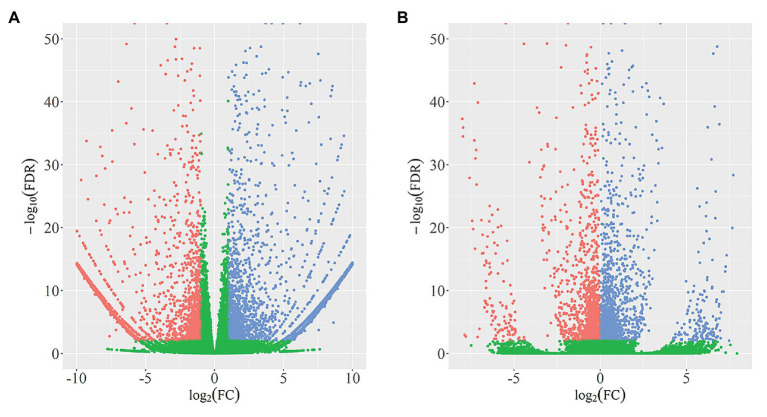
Identification and distribution of differentially expressed genes (DEGs; A) and differential exon usage (DEUs; B). Red dots represent DEGs or DEUs with upregulated expression levels in *qd*, blue dots represent DEGs or DEUs with downregulated expression levels in *qd*, green dots represent genes or exons without significant differences in expression levels between wild-type and *qd*.

**Figure 2:**
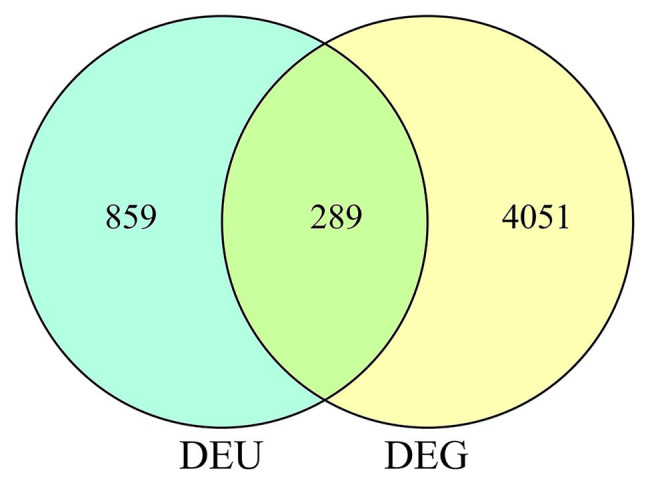
DEUs and DEGs corresponding to differential genes.

In total, we identified 4,340 DEGs (listed in Supplementary Table 2) between wild-type and *qd* plants. Among these genes, 2,462 DEGs showed downregulated expression levels in *qd* than in wild-type plants, and 1878 DEGs showed upregulated expression levels in *qd* than in wild-type plants, as shown in Figure 1A. By using DEXSeq, we identified 2,931 counting bins (listed in Supplementary Table 3), representing 1,148 genes. However, only 289 genes were detected in both DEGs and DEUs, as shown in Figures 1B, 2 and Supplementary Table 4. When we combined the results from DEGs and DEUs, 5,199 genes were identified that could be utilized in GO and KEGG analyses.

### Go Term Analysis

**Figure 3:**
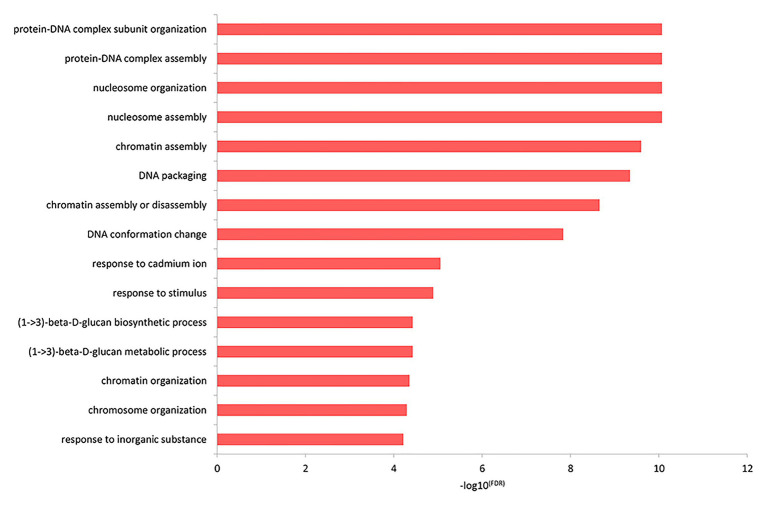
The functional enrichment of the differentially expressed genes in the top GO biological processes.

The top GO biological process enrichment analysis showed that the differentially expressed genes were primarily concentrated in the formation of protein-DNA and other complex macromolecules in the nucleus, as shown in Figure 3 and listed in Supplementary Table 5. The terms included protein-DNA complex subunit organization, protein-DNA complex assembly, nucleosome organization, nucleosome assembly, chromatin assembly, and so on.

**Figure 4:**
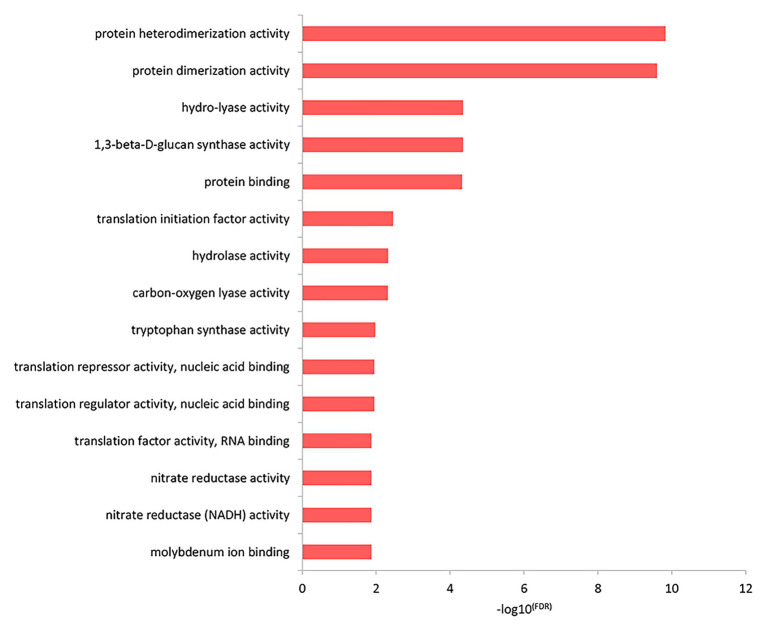
Molecular function enrichment analysis of differentially expressed genes in the top GO terms.

**Figure 5:**
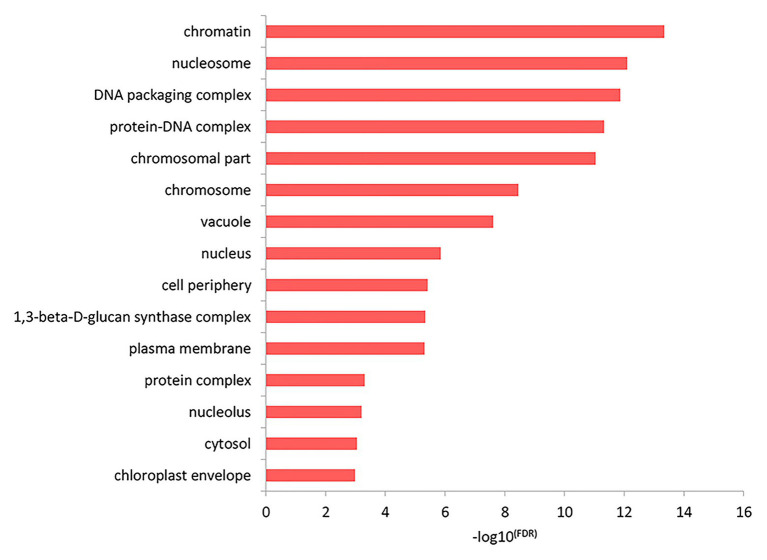
Cell component enrichment analysis of differentially expressed genes in the top GO terms.

The results of the top GO molecular functional enrichment analysis also showed that nucleic acid-protein binding terms were significantly enriched, as shown in Figure 4 and listed in Supplementary Table 5. The top GO cell component enrichment analysis further indicated that chromatin, nucleosomes, and other terms were significantly enriched, as shown in Figure 5 and listed in Supplementary Table 5.

### Benzoxazinoid Biosynthesis Pathway Was Enriched by KEGG Analysis

**Figure 6:**
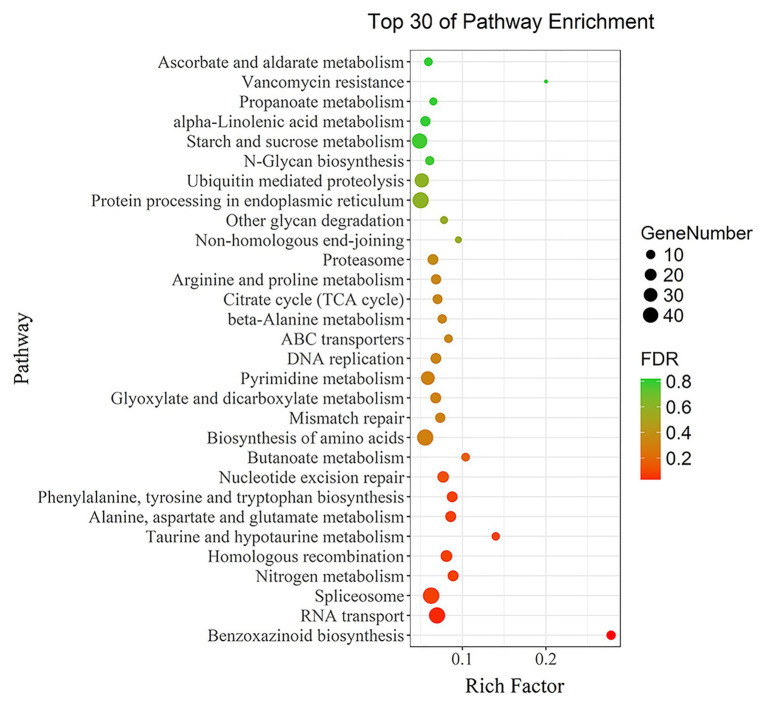
KEGG functional enrichment analysis of differentially expressed genes. Each point in the graph represents a pathway. The size of the spot represents the number of differentially expressed genes enriched in the pathway. The color of the point represents the significance of enrichment, where the red color represents a significantly enriched pathway.

**Figure 7:**
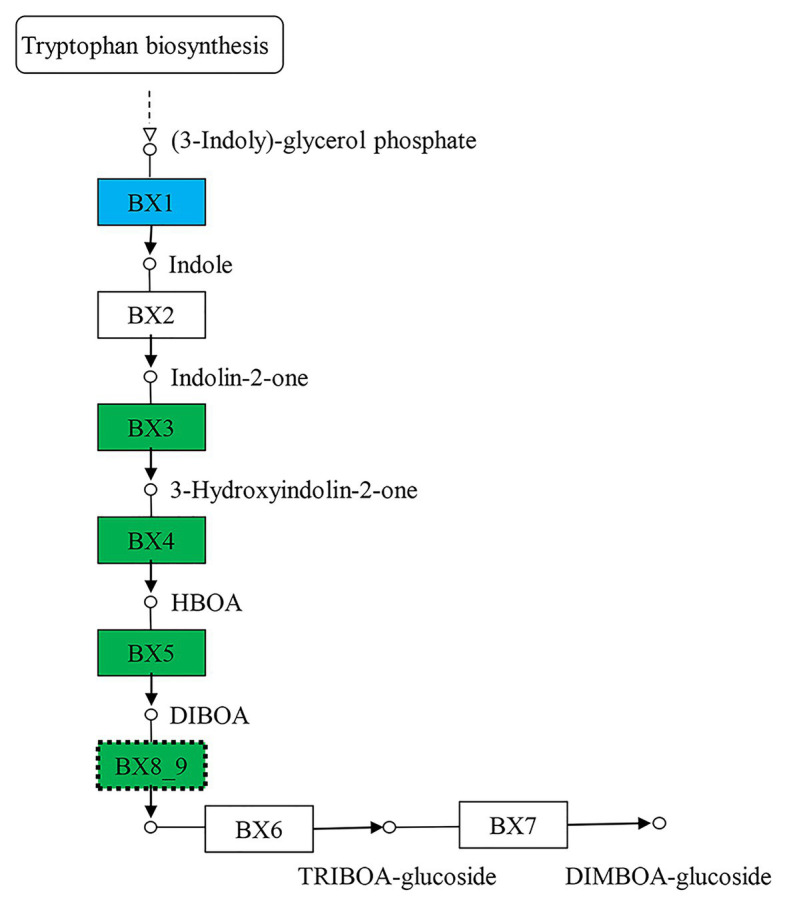
The biosynthesis pathway of benzoxazinoids. The boxes filled with green represent the enzymes containing significantly downregulated DEGs in *qd*. The box filled with blue represents the enzymes that contained both significantly upregulated and downregulated DEGs in *qd*. The dotted boxes represent enzymes containing DEUs.

**Figure 8:**
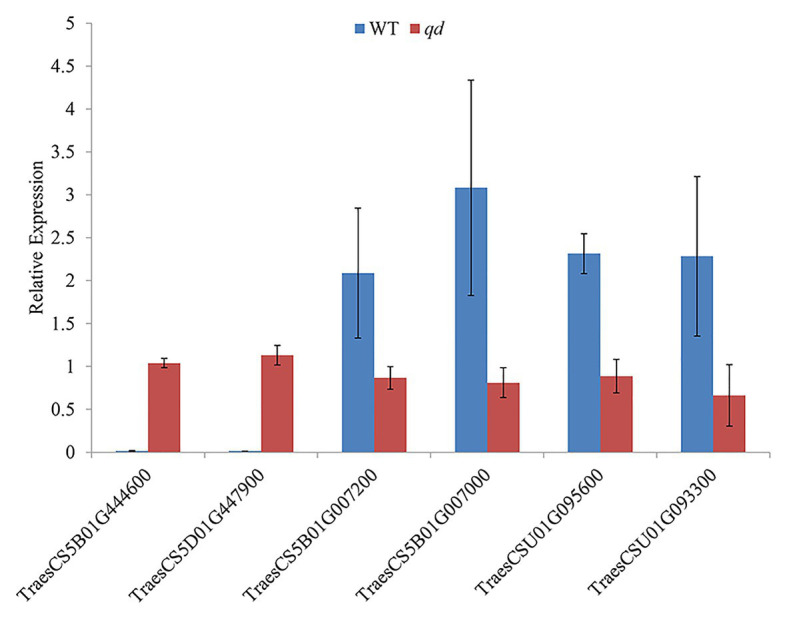
Verification of the DEGs in the benzoxazinoid biosynthesis pathway by qPCR. *Bx1* contained two DEGs, namely, TraesCS5B01G444600 and TraesCS5D01G447900. *Bx3* contained one DEG, namely, TraesCS5B01G007200. *Bx5* contained one DEG, namely, TraesCS5B01G007000. *Bx8_9* contained two DEGs, namely, TraesCSU01G095600 and TraesCSU01G093300.

The 5,199 differentially expressed genes were further analyzed by KEGG enrichment analysis. As shown in Figure 6, nine DEGs and one DEU were significantly enriched in the benzoxazinoid biosynthesis pathway with FDR < 0.01. These nine DEGs and one DEU were distributed among five enzymes of this pathway, namely, *Bx1*, *Bx3*, *Bx4*, *Bx5*, and *Bx8_9*. The *Bx1* gene contained four of these nine DEGs, while *Bx3*, *Bx4*, and *Bx5* contained one of the nine DEGs. The *Bx8_9* gene contained two DEGs and one DEU in this analysis. As shown in Figure 7, the *Bx1* gene contained both upregulated and downregulated DEGs expression, while the DEGs in the other four genes exhibited downregulated expression levels in *qd* plants than in the wild-type. The expression patterns of six of these nine DEGs were successfully verified by qPCR analysis, as shown in Figure 8.

## Discussion

The stems of cereal crops provide mechanical support for lodging resistance and nutrient supply for reproductive organs. The development of crop stems is closely associated with final yield formation (Hirano et al., 2017). Along with stem elongation, inflorescence formation occurs (Gomez-Ariza et al., 2019), which has exhibited great dynamic changes in plant and floret growth-related traits in wheat during SEP (Guo et al., 2018a), and the duration of the SEP influences the number of fertile florets in wheat (Miralles et al., 2000). The wheat mutant *qd* studied in this research was obtained from ion beam mutagenesis, and compared with wild-type plants, the mutant showed an increased stem elongation rate (Zhang et al., 2016). Considerable efforts have been made to obtain a better understanding of the genetic and physical basis of stem elongation regulation in cereal crops. The overexpression of a lectin gene, *OsJAC1*, clearly suppressed rice stem elongation (Jiang et al., 2007). Stem elongation is also regulated by some transcription factors (Xiang et al., 2017; Gomez-Ariza et al., 2019). In recent years, several studies have helped to elucidate the genetic background that affects stem elongation traits, which provides useful information for manipulating stem growth (Borras-Gelonch et al., 2012; Kaur et al., 2017; Guo et al., 2018b). Although we have obtained considerable information on stem elongation regulation, the molecular mechanism governing stem elongation has not been thoroughly elucidated to date. The wheat mutant *qd* may be a useful material to characterize the regulation of stem elongation.

In this study, the benzoxazinoid biosynthesis pathway was identified by transcriptome sequencing analysis, which means that benzoxazinoids may play a role in regulating wheat stem elongation. Benzoxazinoids are traditional crucial elements for crop disease resistance (Ahmad et al., 2011; Tzin et al., 2017). Although benzoxazinoids are considered protective secondary metabolites, secondary metabolites are multifunctional and can function as potent regulators of plant growth (Erb and Kliebenstein, 2020). Benzoxazinoids have been indicated to play a role in improving plant tolerance to soil salinity (Makleit, 2005). In maize, the benzoxazinoid synthesis gene *Bx12* was observed to affect both male and female flowering time (Romero Navarro et al., 2017).

**Figure 9:**
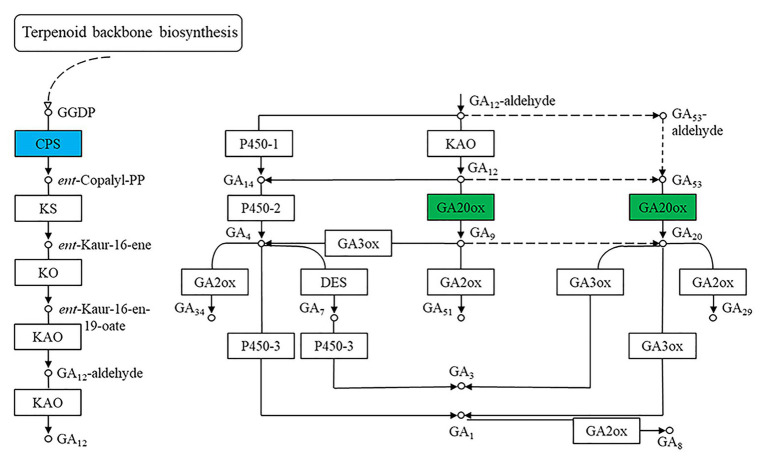
Differentially expressed genes of the gibberellin synthesis pathway. The boxes filled with green represent enzymes containing significantly downregulated DEGs in *qd*. The box filled with blue represents the enzymes that contained significantly upregulated and downregulated DEGs in *qd*.

One way in which benzoxazinoids regulate plant growth and development is through their interactions with plant hormones. Jasmonic acid was proven to regulate benzoxazinoid biosynthesis in plants (Oikawa et al., 2002). Benzoxazinoids and their breakdown products affect auxin-induced plant growth by regulating auxin signaling (Zhou et al., 2018). It has also been demonstrated that benzoxazinoids can inhibit gibberellin-induced α-amylase activity (Kato-Noguchi, 2008). We observed that gibberellins played an important role in regulating the phenotype of the *qd* mutant (Zhang et al., 2016). In this study, we also identified DEGs representing the CPS and GA20ox enzymes, as shown in Figure 9. For the *qd* mutant, the molecular mechanism by which benzoxazinoids are involved in regulating stem elongation in association with the gibberellin signaling pathway remains to be elucidated.

## Data Availability Statement

The datasets presented in this study can be found in online repositories. The names of the repository/repositories and accession number(s) can be found at: https://www.ncbi.nlm.nih.gov/sra/; PRJNA682455.

## Author Contributions

DX did most of the experiments of this study. YX screened the mutant and wrote the manuscript. HG and WZ did some experiments. HX, LZ, JG, SZ, and YD helped in experiments and writing. LL designed this project. All authors contributed to the article and approved the submitted version.

### Conflict of Interest

The authors declare that the research was conducted in the absence of any commercial or financial relationships that could be construed as a potential conflict of interest.

## Footnotes

^1^www.omicshare.com/tools
