# Supplementary material for: Transcriptome Analysis Reveals a Potential Role of Benzoxazinoid in Regulating Stem Elongation in the Wheat Mutant qd
Source: Front Genet. 2021 Feb 9;12:623861. doi: 10.3389/fgene.2021.623861 (PMC7900560; doi:10.3389/fgene.2021.623861)
Supplement: Supplementary file 1 [file Table_1.docx]

The list of primers used for verification

| Gene name | Forward primer sequences (5'-3') | Reverse primer sequences (5'-3') |
| --- | --- | --- |
| TraesCS5B01G444600 | AAGTATCAACGGAGTTAC | TTAGTGACCTGCTTAATC |
| TraesCS5D01G447900 | ATTAAGCAGGTCACTAAC | CGATTATCACTCCATCTG |
| TraesCS5B01G007200 | AGGTTATGTTAGCAAATCTCA | CGACTCAGCCATATCAAC |
| TraesCS5B01G007000 | TCTCACCGACGATAATGT | CAGCACCAGATATGATGTT |
| TraesCSU01G095600 | CTACATGGCGTACCAGACC | TCCACTGGGTCGTCCTTG |
| TraesCSU01G093300 | GAGGATGGCATCGGCGAG | TCAGAATGACTTGATGAGAGCG |
